# Auricular acupressure combined with auricular acupoint massage enhances cognitive function in night shift nurses: a P300 wave analysis

**DOI:** 10.3389/fnhum.2025.1626528

**Published:** 2025-09-30

**Authors:** Xiaoqin Li, Zhun Zhang, Litian Xiao, Xuan Zhang, Huiqi Yao, Fangqin Li, Rongyu Chen, Qian Zhong

**Affiliations:** ^1^Department of Rehabilitation Medicine, Meizhou People’s Hospital, Meizhou, China; ^2^Psychological Clinic of Meizhou People’s Hospital, Meizhou, China; ^3^Department of Comprehensive Geriatric Medicine, The First People’s Hospital of Chenzhou City, Chenzhou, China

**Keywords:** night-shift nurses, cognitive function, auricular acupressure, auricular acupoint massage, P300 wave

## Abstract

**Objectives:**

Night-shift work is associated with cognitive impairments, but convenient, effective, and acceptable traditional Chinese medicine-based interventions remain limited. This study aimed to evaluate the effects of auricular acupressure combined with auricular acupoint massage on cognitive function in night-shift nurses, using P300 wave parameters from electroencephalography analysis as objective metrics.

**Methods:**

Eighty nurses (40 days-shift, 40 night-shift) participated. The intervention included auricular acupressure and massage targeting six points, performed daily for 4 weeks. Cognitive function was assessed using the Insomnia Severity Index (ISI), Montreal Cognitive Assessment (MoCA) and Mini-Mental State Examination (MMSE). P300 amplitude and latency were measured.

**Results:**

Night-shift nurses had significantly higher ISI scores and lower MoCA attention, memory, and total scores compared to day-shift nurses (all *p* < 0.05). Before the intervention, After FDR correction for multiple comparisons, P300 amplitude was significantly lower at the T4 electrode site (q = 0.020) in the night-shift group. P300 latency remained significantly prolonged at sites Fz (q = 0.020), F3 (q < 0.001), F4 (q = 0.035), and T5 (q = 0.033). Post-intervention, the night-shift group demonstrated significant increases in P300 amplitude at F3, F4, T3, T4, T5, and T6 (all q < 0.05) and significant reductions in P300 latency at Fz, F4, F7, T5, and T6 (all q < 0.05). Notably, several sites with affected P300 amplitude and latency before the intervention showed significant improvement following intervention.

**Conclusion:**

Auricular acupressure and massage significantly improved cognitive function in night-shift nurses, evidenced by enhanced P300 parameters. This non-invasive, cost-effective intervention shows promise for alleviating cognitive impairments from shift work.

## 1 Introduction

Nurses working rotating shifts play a critical role in ensuring continuous patient care in healthcare settings. However, the demands of alternating between day and night shifts often disrupt their circadian rhythms, leading to significant cognitive impairments (Al-hrinat et al., 2024; Poormoosa et al., 2024). These impairments typically manifest as reduced alertness, memory difficulties, and slower decision-making speeds (Lin et al., 2024), which not only jeopardize nurses’ physical and mental wellbeing but also increase the likelihood of nursing errors. Such errors can compromise patient safety and the quality of care (Di Muzio et al., 2019). As a result, identifying effective strategies to mitigate cognitive dysfunction in night shift nurses has become an essential focus of both clinical practice and academic research. Night-shift work adversely affects not only nurses but also other healthcare professionals. Reported consequences include increased cardiovascular risk (Yau and Haque, 2019), circadian rhythm disruption (d’Ettorre and Pellicani, 2020), dementia risk (Leso et al., 2021), and broader impacts on health and quality of life (Silva and Costa, 2023). Our study focuses on nurses as a representative group within this broader context.

Current studies on cognitive function in night shift nurses primarily rely on self-report questionnaires or standardized scales (Al-hrinat et al., 2024; Wang et al., 2024). While these methods provide some insight, they are limited by their subjective nature and relatively low sensitivity. For example, Dong et al. (2020) employed multiple instruments, including the State-Trait Anxiety Inventory (STAI), Digit Span Test (DST), and Symbol Digit Modalities Test (SDMT) (Dong et al., 2020). However, such assessments have inherent limitations: they depend on participants’ subjective reporting, which can be influenced by personal perception bias, and many scales use integer-based scoring, restricting their ability to detect subtle or early cognitive changes. To address these limitations, various interventions have been explored to improve sleep and cognitive function in night-shift nurses.

Currently, interventions for sleep and cognitive impairments in night-shift nurses include behavioral measures such as adjusting night-shift schedules, taking daytime naps, and sleep health education, as well as pharmacological treatments targeting sleep-wake regulation (Gurubhagavatula et al., 2021). Behavioral strategies are often difficult to implement effectively and show limited, slow-acting benefits, while pharmacological treatments carry risks of addiction, side effects, and potential drug interactions. These limitations highlight the need for convenient, safe, and effective alternatives such as auricular acupressure and auricular acupoint massage.

Auricular therapy, a key practice in traditional Chinese medicine (TCM), involves stimulating specific points on the ear through techniques such as acupuncture, acupressure, and electrostimulation. This therapy has demonstrated notable efficacy in managing a variety of health issues, particularly in pain relief (Liu et al., 2021), emotional regulation (Lai et al., 2024), and the treatment of sleep disorders (Xia et al., 2023). The therapeutic mechanisms are believed to involve modulation of neurotransmitter secretion and the balance of the endocrine system, thereby enhancing the body’s self-healing abilities (Yang and Xie, 2021; Soltani et al., 2023). Due to its simplicity, minimal side effects, and growing body of evidence supporting its effectiveness, auricular therapy has become an integral part of complementary TCM treatments and is increasingly recognized by modern medical research (Cao et al., 2023).

Event-related potentials (ERP) represent a non-invasive neurophysiological tool used to objectively evaluate cognitive functions by analyzing the brain’s electrical responses to specific stimuli or events. ERP measurements are especially useful in examining changes in attention, memory, and decision-making processes (Boetzel et al., 2024). Among ERP components, the amplitude and latency of the P300 wave are commonly used indicators for assessing cognitive function. P300 latency reflects the speed of stimulus evaluation, increases with task difficulty, and can be elicited by auditory, visual, or somatosensory stimuli (Olichney et al., 2022). P300 amplitude reflects attentional resource allocation (Luck, 2014). Auditory P300 has been used to assess cognitive status in Parkinson’s disease (Rajendran et al., 2023), and both parameters correlate with arithmetic performance (Dickson and Wicha, 2019). Alterations in P300 therefore provide objective markers of cognitive decline, including in night-shift workers (Leso et al., 2021). These metrics have been extensively applied in research on aging, neurodegenerative diseases, and psychiatric disorders (De Salvo et al., 2020; Kaiser et al., 2020; Khan et al., 2022; Olichney et al., 2022). Furthermore, alterations in P300 amplitude and latency have been employed to evaluate the effects of external interventions on cognitive performance, providing objective data to support clinical outcomes (Falkenstein, 2024).

This study aims to investigate the effects of auricular acupressure combined with auricular acupoint massage on cognitive function in night-shift nurses, using P300 latency and amplitude as electrophysiological markers. Day-shift and night-shift nurses underwent baseline assessments of cognitive function and sleep quality using standardized neuropsychological scales (MMSE, MoCA, ISI) and P300 evaluation. Only the night-shift group received the four-week auricular intervention, after which EEG recordings were repeated to assess post-intervention changes. This design allowed comparison between day- and night-shift nurses and evaluation of the potential of auricular therapy to improve cognitive impairments associated with night-shift work.

## 2 Materials and methods

### 2.1 Study design and subjects

This randomized controlled trial was conducted at Meizhou People’s Hospital from January 2023 to September 2023, enrolling a total of 80 nurses: 40 night-shift nurses and 40 day-shift nurses. Participants were randomly assigned to the experimental or control group using a modulo-based randomization approach. This study was conducted as a double-blind randomized controlled trial. Neither the participants nor the practitioners performing the auricular acupressure and massage procedures were aware of the participants’ group assignments. Group allocation was conducted and maintained confidentially by an independent third party (a master’s-level graduate student). The assessors responsible for administering the MoCA, MMSE, and EEG/P300 measurements were also blinded to group assignments to minimize potential experimenter expectancy bias.

The experimental group received auricular acupressure combined with auricular acupoint massage. The control group did not receive any therapeutic intervention but was provided with standardized sleep health education after electroencephalography data collection. The education covered general recommendations, including maintaining a quiet and comfortable sleep environment, avoiding alcohol before bedtime, not overeating or consuming hard-to-digest foods at night, refraining from caffeine or strong tea within 4 h before sleep, engaging in regular physical activity, and avoiding strenuous exercise within 3 h before bedtime.

Day-shift nurses worked regular daytime hours (8 a.m. to 3:30 p.m. or 7:30 a.m. to 11:30 a.m., 2:30 p.m. to 5:30 p.m.) in outpatient clinics without night-shift duties. Night-shift nurses worked rotational schedules in inpatient wards, covering shifts from late afternoon to midnight (3:30 p.m. to 11 p.m.) and/or midnight to early morning (11 p.m. to 8 a.m.).

Inclusion criteria were as follows: (1) nurses actively working in frontline clinical roles (outpatient clinics for day-shift nurses and inpatient wards for night-shift nurses); (2) aged 20–55 years; (3) normal hearing and either normal or corrected-to-normal vision; (4) no history of significant hepatic or renal dysfunction, brain trauma, epilepsy, substance or alcohol abuse, dementia, depression, anxiety, or other psychiatric disorders; (5) a minimum educational level of junior high school; and (6) provision of informed consent. Exclusion criteria were: (1) severe physical disabilities or organic brain conditions requiring electroconvulsive therapy and (2) a history of night-shift work for participants in the day-shift group. Participants were excluded if they failed to cooperate or could not complete the study procedures effectively.

Participants were assigned to groups using a systematic randomization method. Each participant was numbered from 1 to 80, and random numbers were drawn sequentially from a random number table. These numbers were divided by the total number of groups, and the remainders determined group assignments. If the random number was evenly divisible by the group count, the remainder defaulted to the highest group number.

The study protocol was approved by the Medical Ethics Committee of Meizhou People’s Hospital (No. 2022-C-61) and registered in the Medical Research Registration Information System. All participants were provided with detailed information about the study’s objectives, methods, procedures, potential risks, and anticipated benefits. The non-invasive nature and safety of the research were emphasized, and written informed consent was obtained prior to participation.

### 2.2 Baseline psychological and cognitive assessments

After obtaining written informed consent, all participants completed baseline psychological and cognitive assessments prior to the start of the experiment. The assessments included self-administered and clinician-evaluated scales to measure insomnia severity and cognitive function.

The Insomnia Severity Index (ISI) (Morin et al., 2011) was used to evaluate the severity of insomnia over the past 2 weeks. This scale comprises seven items with a total score ranging from 0 to 28. Higher scores indicate more severe insomnia, with clinical severity thresholds defined as follows: 0–4 (no clinically significant insomnia), 5–14 (mild insomnia), 15–21 (moderate insomnia), and 22–28 (severe insomnia).

Cognitive function was assessed using the Mini-Mental State Examination (MMSE) (Folstein et al., 1975) and the Montreal Cognitive Assessment (MoCA) (Nasreddine et al., 2005).

#### 2.2.1 MMSE

This scale evaluates orientation, memory, reading, writing, attention, calculation, recall, naming, and repetition, with a maximum score of 30 points. Correct answers were scored as 1, while incorrect or unanswered items were scored as 0. Given that all participants had at least a junior high school education, a score of 27–30 was considered normal cognitive function, while scores below 22 indicated cognitive impairment.

#### 2.2.2 MoCA

This assessment evaluates multiple cognitive domains, including visuospatial and executive functions, naming, attention, language, abstraction, delayed recall, and orientation. The total score ranges from 0 to 30, with a score of ≥ 26 indicating normal cognitive function and < 26 suggesting impairment.

The MMSE and MoCA assessments were conducted by two intermediate-level psychotherapists with extensive clinical experience to ensure consistency and accuracy. The results of these evaluations were used as baseline data for subsequent analyses.

### 2.3 Auricular acupressure therapy and auricular acupoint massage

**FIGURE 1:**
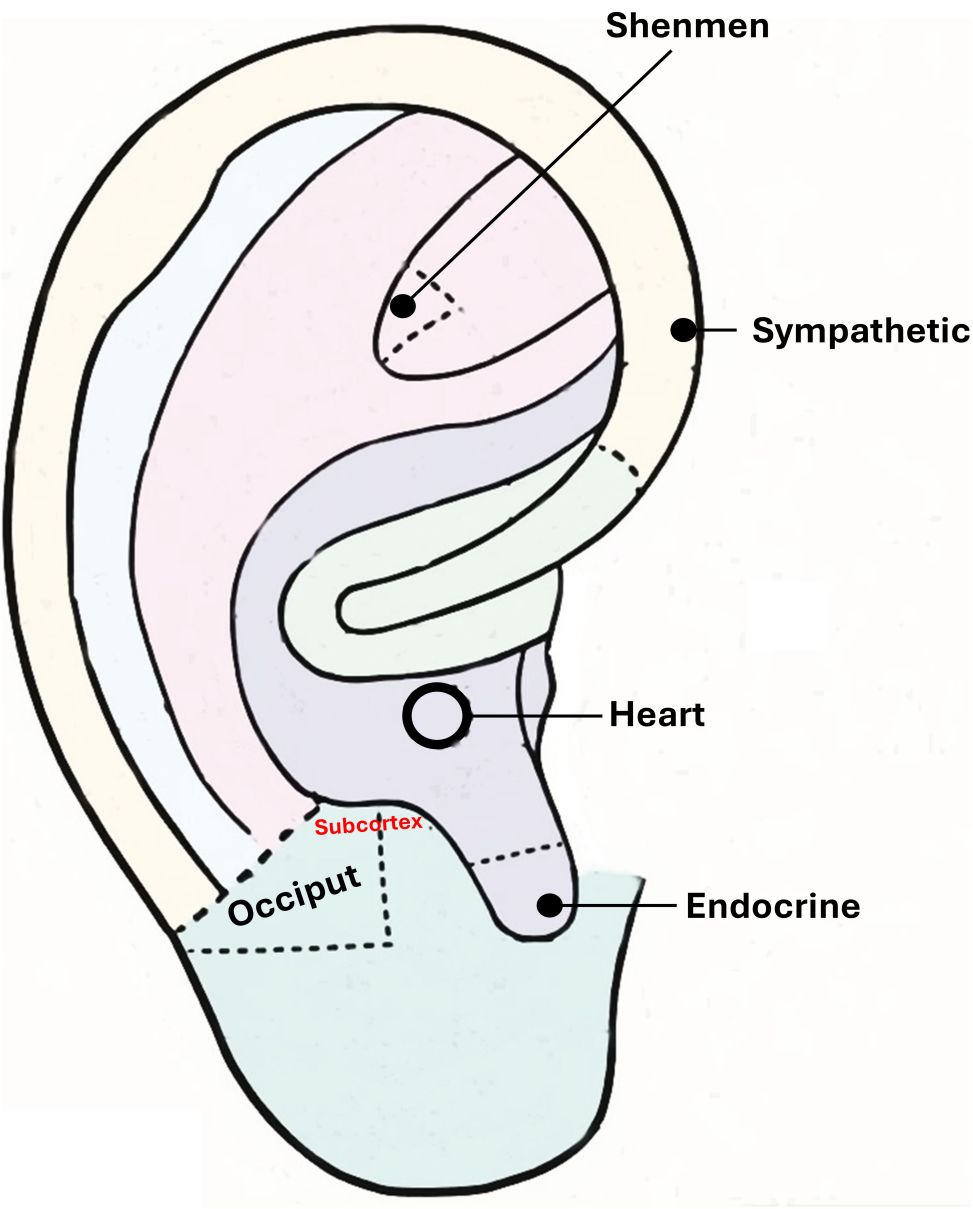
Anatomical locations of the six auricular acupoints used in auricular acupressure and auricular acupoint massage. Points include Heart, Shenmen, Subcortex, Sympathetic, Endocrine, and Occiput.

In auricular acupressure therapy and auricular acupoint massage, specific auricular points are carefully selected to optimize treatment accuracy and therapeutic efficacy. The primary points used include the Heart, Shenmen, Subcortex, Sympathetic, Endocrine, and Occiput points, each with precise anatomical locations (Figure 1). The Heart point is located in the central depression of the cavum concha, while the Shenmen point is situated in the triangular fossa, slightly above the bifurcation of the superior and inferior antihelix crus. The Subcortex point lies on the inner side of the tragus, corresponding to its fourth zone, and the Sympathetic point is positioned at the junction of the terminal end of the inferior antihelix crus and the helix. The Endocrine point is found at the bottom of the cavum concha, approximately 0.5 cm within the intertragic notch, and the Occiput point is located on the outer upper edge of the tragus at the midpoint of its lower border. Depending on the patient’s condition, additional auricular points may be selected by a Traditional Chinese Medicine (TCM) physician following the principles outlined in the “Differential Diagnosis of Traditional Chinese Medicine Symptoms” (Zhao et al., 1984).

Before initiating auricular acupressure therapy, the patient was seated upright to ensure proper posture for the procedure. A thorough examination of the ear skin was conducted to identify any abnormalities, such as damage, swelling, or vascular engorgement. The auricular points and instruments were disinfected using 75% alcohol, which was allowed to evaporate completely before proceeding. Gradual pressure was then applied to the auricular points with appropriate force to identify areas of maximum tenderness, which were subsequently marked with a probe for accurate placement. During the procedure, the auricular point sticking with Vaccaria seeds (manufactured by Wuxi Jiajian Medical Instrument Co., Ltd., Wuxi, China) was aligned with the marked locations. Vertical pressure was applied using the pads of the thumb and index finger, progressively increasing from light to firm until the patient experienced sensations such as soreness, numbness, distension, or pain—responses indicative of the “Deqi” phenomenon. Each auricular point was pressed for 30–60 s, and the process was repeated four times daily at 7:00 a.m., 12:00 p.m., 5:00 p.m., and 10:00 p.m. The Vaccaria seeds were replaced every 3 days throughout the 4 weeks treatment course. To avoid potential damage to the ear skin, lateral or back-and-forth rubbing was avoided during the procedure. Patients were instructed to keep the ear area dry and to prevent dislodgement of the adhesive. In cases where detachment occurred, the adhesive was promptly reapplied to maintain therapeutic efficacy.

Auricular acupoint massage was administered immediately following each auricular acupressure session, constituting an integrated part of the same treatment episode. This combined protocol was performed four times daily throughout the 4 weeks intervention period. Auricular acupoint massage was conducted following a standardized protocol consisting of five techniques: full auricular front-and-back massage, finger-rolling helix massage, ear apex pulling, earlobe kneading, and comprehensive auricular acupoint massage. For the full auricular front-and-back massage, the practitioner first rubbed their palms together until warm, then sequentially pressed and massaged the anterior (front) and posterior (back) surfaces of the auricle until the entire ear became warm. The finger-rolling helix massage involved using the thumb and index finger to roll along the helix, starting from the base, moving upward to the apex, and then forward to the helix root, repeating this motion. The ear apex pulling technique required grasping the upper part of the auricle and gently pulling it upward until the area became warm and hyperemic. Earlobe kneading was performed by pinching the earlobe with the thumb and index finger, pulling downward and outward while simultaneously rubbing the area until hyperemia was achieved. Lastly, the comprehensive auricular acupoint massage targeted specific points starting from the triangular fossa. The massage was performed with the index finger, applying gentle pressure to the cymba concha and cavum concha in sequence, with 20–30 repetitions for each point.

Each session ensured the ear was sufficiently warmed, with noticeable hyperemia and a tingling sensation, to achieve the desired therapeutic effect. To enhance treatment adherence, researchers used a WeChat-based assistant to send reminders and record participants’ compliance with the prescribed techniques. For individuals who did not respond promptly, personalized follow-ups were conducted to ensure the accuracy and safety of the procedures.

### 2.4 Electroencephalography and P300 measures

Cognitive function was assessed using P300 event-related potentials recorded with a medical ERP device (Neuracle Tech. Co., Ltd., Changzhou, China). Electrodes were placed following the international 10–20 system, with A1 and A2 positioned on the left and right mastoids, the reference electrode (CPz), and the grounding electrode (AFz). Vertical and horizontal electrooculogram were recorded simultaneously. The device operated with a bandpass filter range of 0.01–100 Hz and a sampling rate of 1,000 Hz, with electrode impedance maintained below 5 kΩ during data acquisition.

P300 data were acquired from a full set of 20 electrode sites according to the international 10–20 system: Fp1, Fp2, Fz, F3, F4, F7, F8, Cz, C3, C4, T3, T4, Pz, P3, P4, T5, T6, Oz, O1, and O2. An auditory oddball paradigm was employed, featuring a 90 dB auditory stimulus. Target stimuli occurred with a probability of 20%, and non-target stimuli with a probability of 80%. Each target stimulus response was averaged across 1,000 trials. Participants were instructed to remain awake, relaxed, and attentive with their eyes closed while counting target stimuli via a button press. The recording session lasted approximately 20 min, and all EEG data were stored for offline analysis.

The continuous EEG data were preprocessed offline using the instrument’s built-in analysis suite. Data were filtered with a 0.01–100 Hz bandpass and a 50 Hz notch filter. Ocular and muscular artifacts were addressed using Independent Component Analysis (ICA). The EEG was then segmented into epochs from −200 to 1,000 ms relative to stimulus onset. A baseline correction was applied using the −200 ms to 0 ms pre-stimulus period. Epochs containing artifacts with amplitudes exceeding ± 100 μV were automatically excluded from further analysis.

Both P300 latency (ms) and P300 amplitude (μV) were measured from stable event-related potentials. For each participant, the P300 component was identified at each electrode as the most positive peak within a 250–500 ms post-stimulus latency window. P300 amplitude was automatically extracted as the peak amplitude relative to the pre-stimulus baseline. P300 latency was defined as the time from stimulus onset to the occurrence of this peak amplitude.

Participants adhered to strict preparation guidelines before testing. They were required to abstain from alcohol, caffeine, and central nervous system depressants or stimulants for at least 2 weeks. Night-shift and day-shift nurses arrived at the hospital the day before testing and were required to sleep onsite for at least eight hours to standardize conditions. Baseline information, including demographic data and sleep assessments (e.g., ISI), was collected prior to the P300 recordings.

### 2.5 Data collection

Demographic information, psychological and cognitive assessments, and electroencephalography data were collected. Baseline characteristics, including age, sex, education years, and pre-intervention electroencephalography measurements, were recorded for all 80 participants. Post-intervention electroencephalography data, however, were obtained exclusively from the 40 participants working night shifts.

### 2.6 Statistical analysis

The normality of all continuous variables was assessed, and only the age variable met the assumption of normality. Accordingly, age was reported as mean ± standard deviation (SD), while other continuous variables were presented as medians with interquartile ranges (IQR; 25th and 75th percentiles). Comparisons of continuous variables between shift groups were performed using the Mann-Whitney U test, except for age, which was analyzed using Student’s independent *t*-test. The categorical variable, sex, was expressed as counts and percentages and compared using the Chi-square test. Within the night-shift group, pre- and post-intervention electroencephalography results were compared using the Wilcoxon signed-rank test.

EEG data were analyzed across a comprehensive array of 20 electrode sites according to the international 10–20 system. Given that multiple pairwise comparisons were performed for each P300 parameter (amplitude and latency) at these sites, the risk of Type I errors was substantially increased. To account for this multiplicity, the False Discovery Rate (FDR) correction method was applied to the *p*-values derived from the Wilcoxon signed-rank tests (for within-group, pre-post comparisons) and the Mann-Whitney U tests (for between-group comparisons). The FDR correction was implemented using the p.adjust function in R software, version 4.4.1 (R Foundation for Statistical Computing, Vienna, Austria). A corrected q-value of < 0.05 was considered statistically significant for all EEG-based comparisons. Bar charts were utilized to visualize electroencephalography outcomes in the figures. All statistical analyses were conducted using IBM SPSS Statistics, version 25 (IBM Corporation, Somers, NY). The initial *p*-values for EEG comparisons were generated in SPSS, then exported to R specifically for the application of the FDR correction. For all tests other than the EEG comparisons, a two-tailed *p*-value of < 0.05 was considered statistically significant.

## 3 Results

### 3.1 Participant’s baseline characteristics

A total of 80 participants were included in this study, consisting of 10 males (12.50%) and 70 females (87.50%). The average age was 34.54 ± 8.07 years, with a median of 10 years of education. Forty participants worked the day shift, while 40 worked the night shift. The baseline characteristics of the participants are summarized in Table 1. There were no significant differences between the two groups in terms of age, sex, education level, MMSE, or several MoCA subdomains (all *p* > 0.05). However, the night-shift participants had higher ISI scores and lower scores on MoCA attention, memory, and total scores (all *p* < 0.05).

**TABLE 1:** Participant’s baseline characteristics by shift groups.

Parameters	Day shift (n = 40)	Night shift (n = 40)	All (n = 80)	*P*
Age	34.88 ± 7.76	34.20 ± 8.46	34.54 ± 8.07	0.711
Sex				0.176
Male	3 (7.50%)	7 (17.50%)	10 (12.50%)	
Female	37 (92.50%)	33 (82.50%)	70 (87.50%)	
Education year	14 (13, 15)	15 (13, 16)	15 (13, 15.75)	0.275
ISI score	12 (8.25, 14.75)	18 (16, 23.75)	15.50 (11, 20)	< 0.001
MMSE	28 (27, 29)	28 (27, 29)	28 (27, 29)	0.263
**MoCA**				
Visuospatial and executive functions	5 (4, 5)	5 (4, 5)	5 (4, 5)	0.811
Naming	3 (3, 3)	3 (3, 3)	3 (3, 3)	0.317
Attention	5 (5, 6)	5 (4, 5)	5 (5, 5)	< 0.001
Language	3 (3, 3)	3 (3, 3)	3 (3, 3)	1.000
Abstraction	2 (2, 2)	2 (2, 2)	2 (2, 2)	0.317
Memory	5 (4, 5)	4 (4, 5)	4 (4, 5)	< 0.01
Orientation	6 (5, 6)	6 (5, 6)	6 (5, 6)	0.650
Total	28 (27.25, 29)	27 (27, 28)	28 (27, 28)	< 0.001

### 3.2 Pre-intervention electroencephalography results

Before the intervention, P300 wave parameters, including amplitude and latency across various brain regions, were measured for all participants. Table 2 summarizes the pre-intervention electroencephalography results for both the day-shift and night-shift groups, providing P300 amplitude and latency data.

**TABLE 2:** Participant’s pre-intervention electroencephalography results.

Parameters	Day shift (n = 40)	Night shift (n = 40)	All (n = 80)	*P*	Adjusted *q* (FDR)
**P300 amplitude**					
Fp1	0.40 (−1.48, 1.98)	1.95 (−0.78, 3.78)	1.05 (−0.80, 2.95)	0.057	0.285
Fp2	1.50 (−1.15, 2.75)	1.55 (−0.20, 3.10)	1.50 (−0.40, 3.08)	0.637	0.749
Fz	1.50 (0.08, 3.90)	0.70 (0.20, 1.30)	0.80 (0.20, 1.93)	0.047	0.285
F3	1.95 (0.48, 4.63)	1.30 (0.23, 2.08)	1.40 (0.30, 2.58)	0.037	0.285
F4	1.65 (0.43, 4.40)	1.05 (0.60, 1.68)	1.25 (0.53, 2.88)	0.117	0.346
F7	2.15 (−0.18, 5.18)	2.25 (1.45, 4.65)	2.20 (−0.03, 4.78)	0.740	0.779
F8	2.70 (0.48, 5.50)	2.35 (0.65, 4.38)	2.65 (0.63, 4.80)	0.516	0.688
Cz	1.45 (0.13, 5.43)	1.55 (1.30, 2.30)	1.55 (0.90, 2.95)	0.573	0.716
C3	2.35 (0.33, 4.80)	2.00 (1.23, 2.90)	2.05 (0.73, 3.18)	0.733	0.778
C4	1.40 (0.33, 4.28)	1.65 (1.03, 2.38)	1.60 (0.83, 3.00)	0.885	0.885
T3	2.95 (2.23, 4.58)	3.10 (0.73, 4.13)	3.10 (2.10, 4.28)	0.231	0.462
T4	3.45 (2.63, 5.05)	2.50 (2.03, 3.18)	2.95 (2.23, 3.90)	0.001	0.020
Pz	2.00 (0.55, 5.00)	1.70 (0.33, 3.60)	1.80 (0.43, 4.18)	0.368	0.583
P3	2.40 (1.10, 4.98)	1.85 (0.70, 3.38)	2.20 (0.70, 4.58)	0.206	0.458
P4	2.85 (0.73, 5.00)	2.50 (0.60, 3.98)	2.60 (0.63, 4.28)	0.413	0.590
T5	3.20 (2.80, 5.30)	3.25 (1.23, 5.30)	3.20 (2.03, 5.30)	0.176	0.440
T6	3.20 (2.43, 4.50)	3.00 (1.55, 4.28)	3.10 (1.83, 4.48)	0.102	0.346
Oz	2.30 (−0.03, 4.85)	3.50 (2.53, 5.18)	3.25 (1.33, 5.08)	0.121	0.346
O1	2.85 (−0.18, 4.73)	3.20 (1.43, 4.98)	3.05 (0.60, 4.88)	0.379	0.583
O2	2.55 (0.38, 5.13)	2.95 (2.00, 4.98)	2.75 (1.30, 4.98)	0.305	0.555
**P300 latency**					
Fp1	336.50 (327.50, 350.50)	345.00 (322.25, 372.50)	340.00 (324.25, 359.25)	0.187	0.197
Fp2	330.00 (313.50, 355.25)	342.00 (322.50, 366.00)	337.00 (316.25, 358.75)	0.103	0.129
Fz	331.50 (311.00, 349.75)	351.00 (331.25, 385.25)	343.00 (320.00, 363.75)	0.002	0.020
F3	313.00 (301.00, 351.00)	341.50 (326.25, 382.00)	335.50 (312.00, 368.00)	< 0.001	< 0.001
F4	328.00 (305.00, 347.50)	351.00 (322.25, 378.75)	340.00 (312.50, 367.25)	0.007	0.035
F7	328.00 (306.25, 350.50)	342.00 (319.75, 384.75)	338.50 (311.25, 360.25)	0.031	0.088
F8	342.50 (312.00, 369.50)	345.00 (311.25, 381.75)	342.50 (312.00, 380.00)	0.470	0.470
Cz	326.00 (302.25, 358.25)	352.50 (311.25, 369.25)	341.00 (307.00, 361.00)	0.082	0.129
C3	340.00 (320.00, 356.25)	356.00 (325.00, 386.25)	345.50 (321.25, 379.50)	0.091	0.129
C4	342.50 (328.25, 356.00)	364.50 (311.25, 388.00)	351.00 (326.50, 376.75)	0.077	0.129
T3	336.50 (310.25, 359.25)	353.00 (326.50, 380.75)	342.50 (316.25, 376.00)	0.030	0.088
T4	341.50 (319.50, 362.50)	364.00 (320.25, 388.00)	347.00 (320.25, 378.00)	0.059	0.129
Pz	342.00 (308.50, 374.25)	370.00 (312.50, 412.00)	351.00 (310.25, 386.25)	0.065	0.129
P3	345.50 (330.25, 364.00)	370.50 (327.25, 384.00)	352.00 (330.25, 378.75)	0.035	0.088
P4	342.50 (320.00, 364.00)	370.50 (312.00, 387.75)	349.00 (319.00, 383.00)	0.141	0.166
T5	343.00 (313.25, 359.50)	373.00 (329.00, 400.00)	349.50 (317.00, 379.75)	0.005	0.033
T6	340.50 (313.00, 354.50)	368.50 (327.00, 388.00)	349.00 (317.00, 380.00)	0.018	0.072
Oz	348.00 (338.50, 365.75)	371.00 (336.00, 389.25)	350.00 (338.00, 376.00)	0.103	0.129
O1	346.00 (339.25, 359.75)	365.00 (321.75, 376.00)	348.00 (336.75, 370.00)	0.186	0.197
O2	348.00 (330.00, 376.50)	370.50 (337.75, 387.00)	358.00 (333.50, 380.00)	0.095	0.129

After FDR correction for multiple comparisons across the 20 electrode sites, the results were as follows:

For P300 amplitude, participants in the night-shift group exhibited significantly lower value only at the T4 electrode site (q = 0.020) compared to the day-shift group.

In terms of P300 latency, the night-shift group demonstrated significantly prolonged latency at the Fz (q = 0.020), F3 (q < 0.001), F4 (q = 0.035), and T5 (q = 0.033) electrode sites.

**FIGURE 2:**
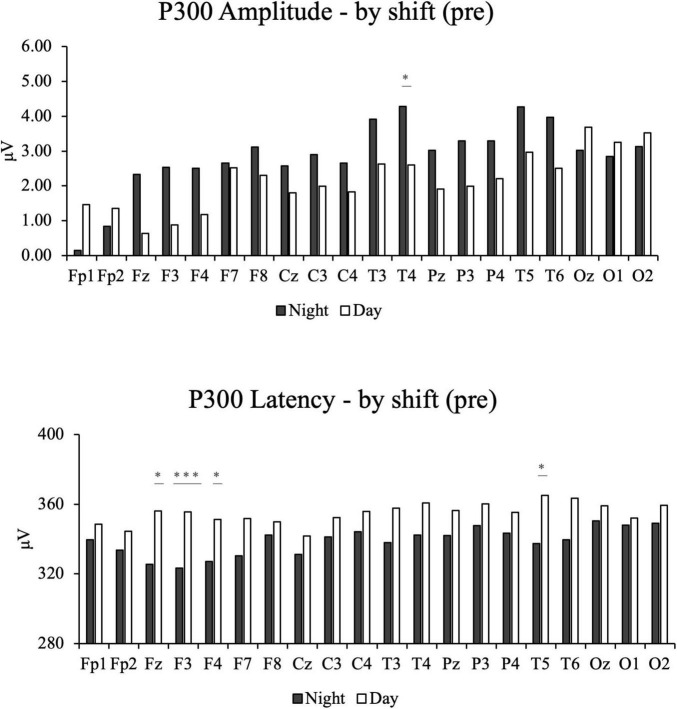
The bar chats of participants’ pre-intervention electroencephalography results by night and day shifts, including P300 amplitude and P300 Latency. *Indicates that the adjusted q-value (FDR) is less than 0.05. ***Indicates that the adjusted q-value (FDR) is less than 0.001.

**FIGURE 3:**
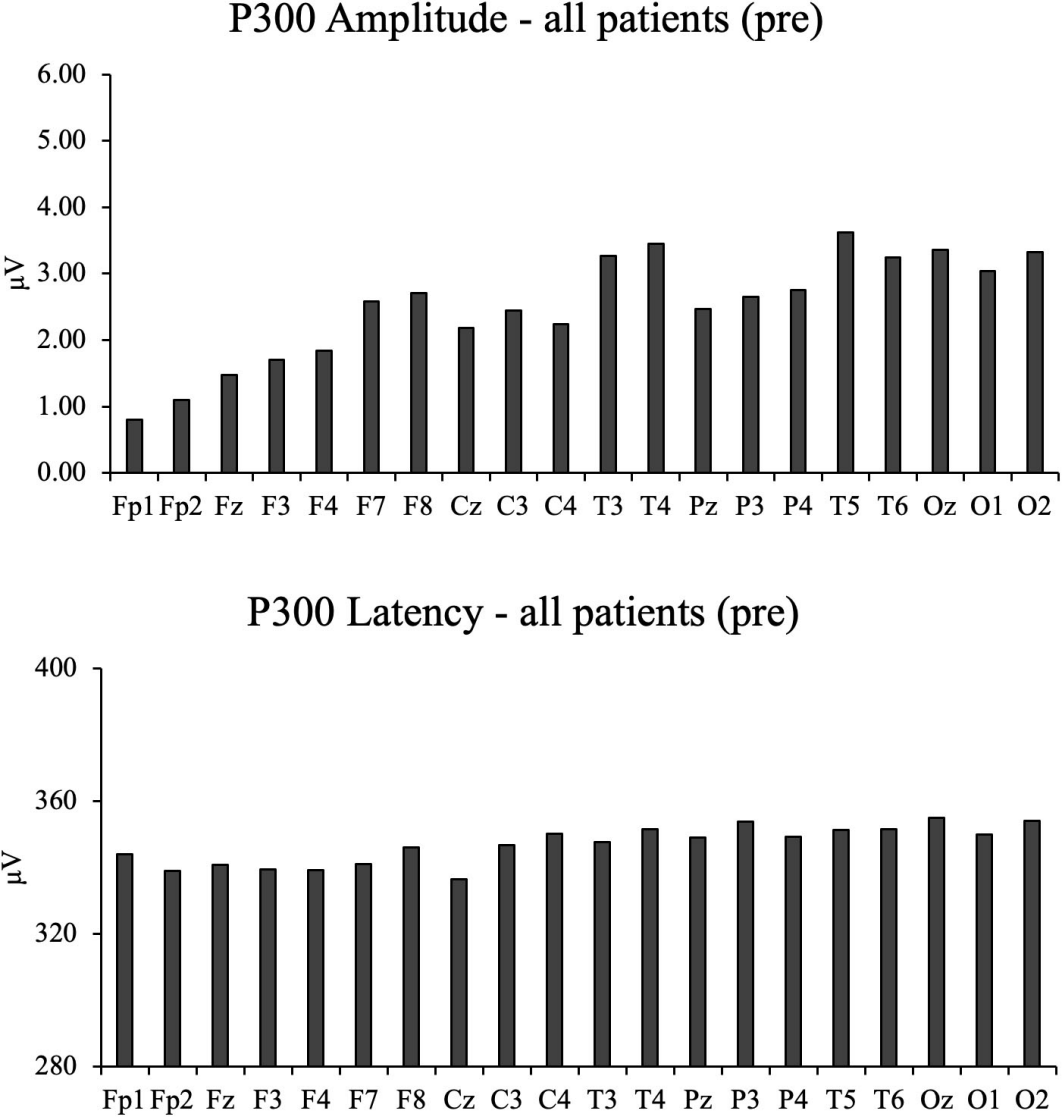
The bar charts of all participant’s pre-intervention electroencephalography results, including P300 amplitude and P300 Latency.

These findings are visually represented in Figure 2, where statistically significant electrode sites are marked with asterisks. Figure 3 illustrates the overall distribution of P300 amplitudes and latencies across all participants.

### 3.3 Comparisons between pre- and post-intervention results in the night-shift group

In the night-shift group, post-intervention P300 wave parameters were collected and compared with pre-intervention data. Table 3 summarizes the electroencephalographic results, including P300 amplitude and latency for the night-shift group.

**TABLE 3:** The pre- and post-intervention electroencephalography results in night-shift group.

Parameters	Night shift (n = 40)		Change (post minus pre)	*P*	Adjusted *q* (FDR)
	Pre	Post			
**P300 amplitude**					
Fp1	1.95 (−0.78, 4.25)	2.10 (−0.85, 4.38)	0.15 (0.00, 0.45)	0.030	0.075
Fp2	1.55 (−0.20, 3.10)	1.45 (−0.10, 3.28)	0.05 (−0.05, 0.10)	0.122	0.203
Fz	0.70 (0.20, 1.30)	0.70 (0.03, 1.38)	0.10 (0.00, 0.20)	0.028	0.075
F3	1.30 (0.23, 2.08)	1.35 (0.23, 2.20)	0.15 (0.05, 0.25)	0.001	0.005
F4	1.05 (0.60, 1.68)	1.15 (0.63, 2.10)	0.15 (0.05, 0.25)	0.001	0.005
F7	2.25 (1.45, 4.65)	2.40 (1.50, 4.78)	0.05 (0.00, 0.10)	0.082	0.164
F8	2.35 (0.65, 4.38)	2.50 (0.68, 4.50)	0.05 (0.00, 0.15)	0.077	0.164
Cz	1.55 (1.30, 2.30)	1.60 (1.13, 2.28)	0.25 (−0.05, 0.10)	0.485	0.544
C3	2.00 (1.23, 2.90)	2.20 (1.15, 2.95)	0.05 (0.00, 0.10)	0.164	0.252
C4	1.65 (1.03, 2.38)	1.75 (0.93, 2.28)	0.05 (−0.05, 0.10)	0.490	0.544
T3	3.10 (0.73, 4.13)	3.20 (1.08, 4.28)	0.15 (0.05, 0.25)	0.001	0.005
T4	2.50 (2.03, 3.18)	2.60 (2.23, 3.30)	0.15 (0.05, 0.25)	0.002	0.008
Pz	1.70 (0.33, 3.60)	1.90 (0.23, 3.45)	0.00 (−0.05, 0.10)	0.406	0.539
P3	1.85 (0.70, 3.38)	1.70 (0.83, 3.35)	0.00 (−0.10, 0.10)	0.815	0.815
P4	2.50 (0.60, 3.98)	2.60 (0.63, 4.05)	0.00 (−0.05, 0.10)	0.609	0.641
T5	3.25 (1.23, 5.30)	3.50 (1.50, 5.28)	0.20 (0.10, 0.25)	< 0.001	0.005
T6	3.00 (1.55, 4.28)	3.00 (1.60, 4.48)	0.15 (0.05, 0.25)	0.004	0.013
Oz	3.50 (2.53, 5.18)	3.45 (2.58, 5.20)	0.00 (−0.05, 0.10)	0.431	0.538
O1	3.20 (1.43, 4.98)	3.15 (1.35, 5.10)	0.05 (0.00, 0.10)	0.100	0.182
O2	2.95 (2.00, 4.98)	2.90 (2.20, 5.03)	0.05 (−0.05, 0.10)	0.374	0.534
**P300 latency**					
Fp1	345.00 (322.25, 372.50)	341.50 (318.50, 369.50)	−3.00 (−6.50, 0.00)	0.065	0.155
Fp2	342.00 (322.50, 366.00)	340.50 (319.25, 358.75)	−2.50 (−5.50, 0.50)	0.116	0.211
Fz	351.00 (331.25, 385.25)	347.50 (320.00, 370.50)	−7.50 (−13.00, −3.00)	0.004	0.020
F3	341.50 (326.25, 382.00)	342.50 (316.25, 391.75)	−5.00 (−9.50, −0.50)	0.028	0.080
F4	351.00 (322.25, 378.75)	343.50 (319.00, 367.00)	−8.50 (−14.00, −4.00)	0.001	0.010
F7	342.00 (319.75, 384.75)	336.50 (315.00, 365.75)	−8.00 (−12.50, −4.00)	0.001	0.010
F8	345.00 (311.25, 381.75)	345.50 (310.75, 380.75)	−2.00 (−5.50, 2.00)	0.273	0.364
Cz	352.50 (311.25, 369.25)	341.50 (310.25, 371.75)	−1.00 (−4.00, 2.50)	0.576	0.606
C3	356.00 (325.00, 386.25)	355.50 (328.50, 385.00)	1.00 (−2.00, 4.00)	0.542	0.602
C4	364.50 (311.25, 388.00)	366.50 (316.50, 384.00)	−1.50 (−5.00, 1.50)	0.273	0.364
T3	353.00 (326.5, 380.75)	351.00 (330.25, 382.75)	−6.00 (−10.50, −1.00)	0.019	0.063
T4	364.00 (320.25, 388.00)	361.50 (317.50, 392.50)	−4.25 (−9.00, 1.00)	0.070	0.156
Pz	370.00 (312.5, 412.00)	372.50 (313.25, 404.00)	−2.00 (−5.00, 1.50)	0.231	0.364
P3	370.5 (327.25, 384.00)	373.50 (331.25, 385.00)	−1.00 (−4.00, 2.50)	0.489	0.585
P4	370.50 (312.00, 387.75)	368.50 (315.25, 382.5)	−1.00 (−5.00, 2.00)	0.497	0.585
T5	373.00 (329.00, 400.00)	365.50 (319.50, 391.75)	−7.50 (−11.50, −2.50)	0.002	0.013
T6	368.50 (327.00, 388.00)	361.00 (320.00, 388.00)	−6.00 (−10.00, −1.50)	0.005	0.020
Oz	371.00 (336.00, 389.25)	361.50 (329.25, 385.75)	−2.00 (−5.50, 1.50)	0.258	0.364
O1	365.00 (321.75, 376.00)	362.00 (325.25, 378.50)	−0.50 (−4.50, 3.50)	0.775	0.775
O2	370.50 (337.75, 387.00)	363.50 (328.50, 384.75)	−2.50 (−6.00, 0.50)	0.104	0.208

Following FDR correction, for P300 amplitude, significant increases were observed at the F3 (q = 0.005), F4 (q = 0.005), T3 (q = 0.005), T4 (q = 0.008), T5 (q = 0.005), and T6 (q = 0.013) electrode sites.

**FIGURE 4:**
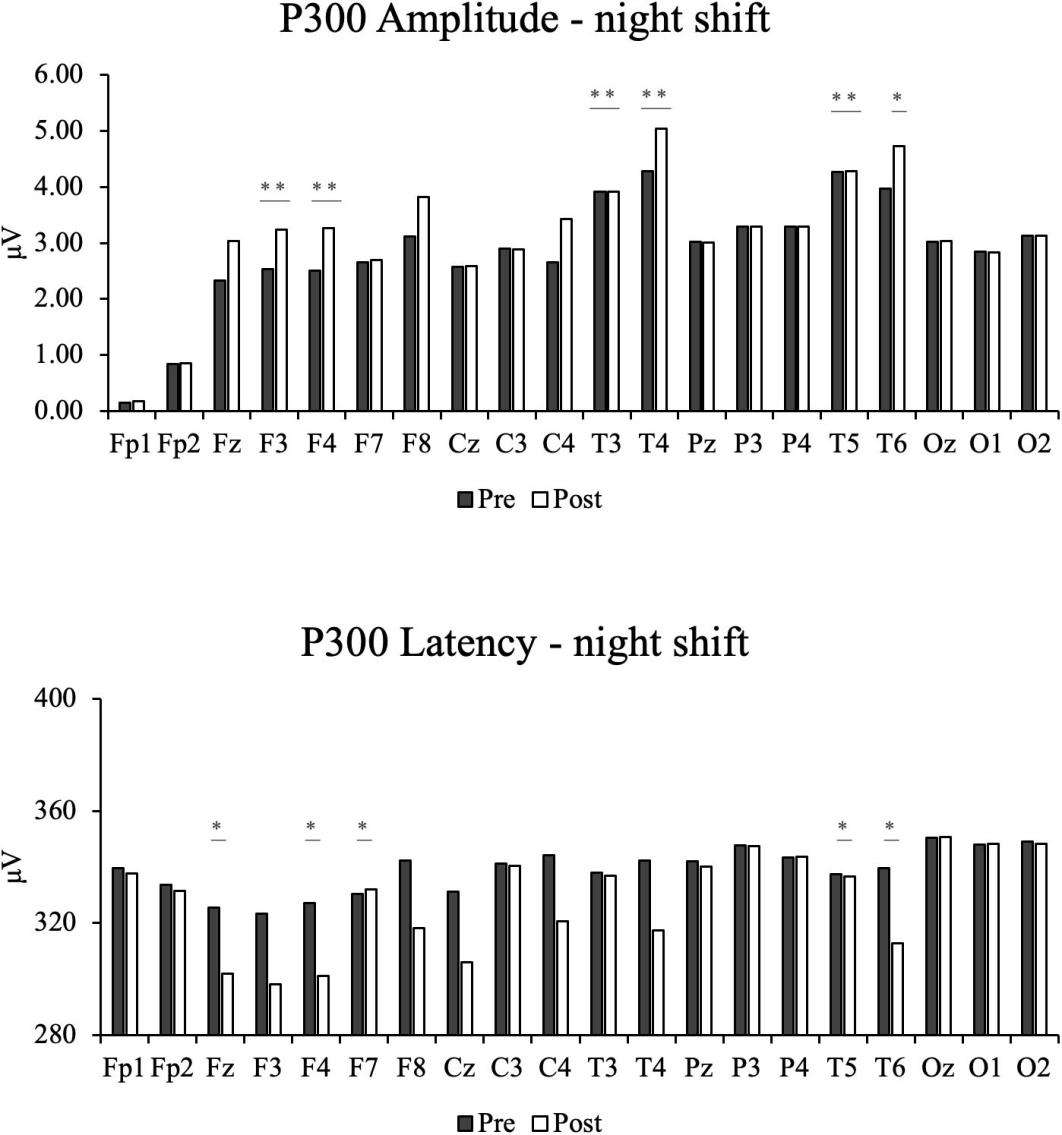
The bar chats of participants’ post-intervention electroencephalography results in the night shift group, including P300 amplitude and P300 Latency. *Indicates that the adjusted q-value (FDR) is less than 0.05. ***Indicates that the adjusted q-value (FDR) is less than 0.001.

Conversely, for P300 latency, post-intervention values were significantly lower at Fz (q = 0.020), F4 (q = 0.010), F7 (q = 0.010), T5 (q = 0.013), and T6 (q = 0.020) electrode sites. Overall, the intervention led to a notable increase in P300 amplitude and a decrease in P300 latency, with significant changes occurring predominantly in the frontal and temporal regions. These findings are also illustrated in Figure 4.

## 4 Discussion

This study investigated the impact of night-shift work on participants’ psychological and cognitive functions, incorporating P300 measurements to provide an objective perspective on the sleep quality and cognitive performance of night-shift workers. As shown in Table 1, results from the ISI and MoCA evaluations revealed significant declines in sleep quality and cognitive ability in the night-shift group. Specifically, the ISI scores of the night-shift group were significantly higher than those of the day-shift group (*P* < 0.001), indicating more severe insomnia among night-shift workers (Yook et al., 2024). This disparity suggests that night-shift work may lead to sleep deprivation or reduced sleep quality, consequently impairing cognitive function. Regarding cognitive performance, the night-shift group scored significantly lower on the MoCA (Montreal Cognitive Assessment) across multiple dimensions, particularly in attention (*P* < 0.001) and memory (*P* < 0.01). Additionally, the total MoCA scores of the night-shift group were notably lower than those of the day-shift group (*P* < 0.001). These findings indicate that night-shift work significantly impairs cognitive functions, particularly executive function and memory (Bufano et al., 2024).

To further validate the objectivity of the subjective evaluations, ERP P300 waveform measurements were utilized to provide a more detailed analysis of participants’ neurocognitive function. It is important to note that all reported significant findings for EEG comparisons are based on FDR correction for multiple comparisons across the 20 electrode sites. This rigorous statistical approach ensures that our reported results are robust and not due to chance findings, thereby strengthening the validity of our conclusions regarding the intervention’s effects on specific neural circuits. The P300 results revealed significantly reduced amplitudes in the night-shift group at the T4 electrode site (q = 0.020). Although uncorrected p-values were significant at Fz and F3, these differences did not survive FDR correction for multiple comparisons (q = 0.285 for both). The reduced amplitude at T4 reflects diminished cognitive processing efficiency in this temporal region (Juan et al., 2019). A decrease in P300 amplitude is generally associated with reduced attentional resources or impaired cognitive performance, suggesting diminished neural resource allocation and cognitive engagement during tasks in the night-shift group (Lima et al., 2022). Furthermore, after FDR correction, prolonged P300 latencies remained significant in the night-shift group at the Fz (q = 0.020), F3 (q < 0.001), F4 (q = 0.035), and T5 (q = 0.033) electrode sites. These delays indicate slower neural processing speeds, particularly in the frontal and temporal regions. Increased P300 latency reflects prolonged information processing time, leading to slower cognitive response speeds and delayed reactions to stimuli (Jones et al., 2024). These P300 findings provide biological support for the subjective assessments and shed light on potential neural mechanisms underlying the observed cognitive deficits.

These results are consistent with a body of existing literature on P300 as a marker of cognitive function. Previous studies have documented reduced P300 amplitudes and prolonged latencies in night-shift workers, a pattern consistent with impaired attention, memory, and slowed cognitive processing (Juan et al., 2019; Lima et al., 2022; Jones et al., 2024). Night-shift work disrupts circadian rhythms (d’Ettorre and Pellicani, 2020), which in turn predisposes individuals to sleep and biological-rhythm disturbances (Silva and Costa, 2023), thereby impeding cognitive recovery and increasing the risk of long-term cognitive decline including dementia (Leso et al., 2021). Our results align with and extend these reports by precisely identifying the specific frontal and temporal brain regions most affected by shift work through multi-electrode analysis, further solidifying the utility of P300 as an objective electrophysiological marker of shift-work-related cognitive impairment.

These results suggest that long-term night-shift work significantly affects higher-order cognitive functions, particularly in the frontal and temporal regions of the brain. Chronic sleep deprivation and irregular work schedules may disrupt circadian rhythms, impairing the normal functioning of the nervous, immune, and endocrine systems, ultimately resulting in decreased cognitive resource allocation and slower neural responses (Garbarino et al., 2021; Sullan et al., 2021; Díaz-Del Cerro et al., 2022). This study demonstrated poorer performance in multiple psychological and cognitive assessment dimensions among night-shift participants, particularly in reaction time and memory, which could further reduce learning and work efficiency. The findings are consistent with existing literature highlighting the long-term adverse effects of night-shift work on brain health, underscoring the generalizability and reliability of this study’s conclusions.

Current management strategies for shift-work-related cognitive decline primarily include pharmacological interventions (e.g., melatonin, modafinil) and behavioral modifications (e.g., sleep hygiene education, controlled light exposure) (Gurubhagavatula et al., 2021). While beneficial, these approaches can be limited by potential side effects, contraindications, or challenges in long-term adherence. It is within this context that non-invasive, complementary approaches like auricular therapy require further exploration.

Given the significant cognitive impairments observed in night-shift workers, auricular therapy was implemented as a potential intervention to mitigate these effects (Ovliaei Bidgoli et al., 2024). After auricular acupressure and auricular acupoint massage treatment, the night-shift group showed significant increases in P300 amplitude at the F3 (q = 0.005), F4 (q = 0.005), T3 (q = 0.005), T4 (q = 0.008), T5 (q = 0.005), and T6 (q = 0.013) electrode sites after FDR correction. Notably, the T4 site, which exhibited a significantly reduced amplitude before the intervention (q = 0.020), showed marked improvement in amplitude (q = 0.008). Additionally, sites F3 and F4, which showed prolonged latency pre-intervention, showed significant shortening in latency post-intervention. Similarly, post-treatment P300 latency values were significantly shortened at Fz (q = 0.020), F4 (q = 0.010), F7 (q = 0.010), T5 (q = 0.013), and T6 (q = 0.020). These results indicate that auricular acupressure and auricular acupoint massage effectively enhanced P300 amplitude and shortened latency, potentially alleviating neurocognitive deficits in multiple brain regions. Importantly, our finding that this combined intervention increased P300 amplitude and shortened latency suggests that the electrophysiological deficits associated with night-shift work are at least partially reversible with a timely, non-pharmacological intervention. Similar findings have been reported in previous studies, which demonstrated that acupuncture improved P300 amplitude and shortened latency more effectively than Western medicine or cognitive rehabilitation training alone, highlighting its potential for cognitive function rehabilitation (Li et al., 2022). Notably, the intervention appears to boost brain activity in the frontal and temporal lobes, potentially improving higher cognitive functions such as reaction time and processing speed.

Given its low cost, non-invasive nature, and ease of implementation, the auricular therapy protocol used in this study may represent a feasible and attractive complementary or alternative strategy to mitigate cognitive deficits in night-shift populations. These promising results warrant further evaluation in larger, controlled trials.

This study has several limitations that should be acknowledged. First, the sample size was relatively small, with only 80 participants, which may limit the generalizability of the findings. Second, the study was conducted at a single healthcare institution, potentially introducing institutional biases that may not represent broader populations. Third, the absence of a placebo or sham control group limits the ability to distinguish the specific therapeutic effects of auricular acupressure and massage from potential placebo or expectancy effects. Fourth, although our study identified electrode-specific changes in P300 amplitude and latency, we were unable to provide topographical scalp maps because the ERP recording equipment was on a trial basis and was retrieved by the manufacturer after the study, preventing further data reprocessing and visualization. Finally, the study’s short follow-up period does not allow for the assessment of long-term efficacy and sustainability of the intervention. Future research should confirm these findings through larger, multicenter trials, incorporate sham auricular interventions to establish specificity, apply advanced ERP mapping to elucidate spatial patterns, and evaluate the long-term effects of auricular therapy. This study highlights the significant impact of night-shift work on cognitive function, particularly in areas related to reaction time and processing speed, as evidenced by impaired P300 amplitude and latency in key brain regions. Through the application of auricular acupressure therapy combined with auricular acupoint massage, we observed marked improvements in P300 parameters, suggesting enhanced cognitive processing and response efficiency in night-shift nurses. These findings underscore the potential of this intervention as a simple, safe, and cost-effective strategy for mitigating cognitive impairments associated with shift work. This study provides an objective tool for assessing cognitive function and introduces a promising intervention to improve the cognitive performance and well-being of night-shift nurses, potentially enhancing patient care and advancing evidence-based practices in occupational health.

## Data Availability

The original contributions presented in this study are included in this article/supplementary material, further inquiries can be directed to the corresponding author.
